# *Pimpinella brachycarpa* extract attenuates periodontal inflammation by modulating the PI3K/Akt–NF-κB signaling pathway

**DOI:** 10.3389/fphar.2026.1902876

**Published:** 2026-09-29

**Authors:** A. Yeong Lee, Su-Bin Park, Eunjung Son, Junghyun Kim, Dong-Seon Kim, Soon yeong Park, Hyeri Ku, Jung-In Kim, Yun Mi Lee

**Affiliations:** 1 KM Data Division, Korea Institute of Oriental Medicine, Daejeon, Republic of Korea; 2 Department of Oral Pathology, School of Dentistry, Jeonbuk National University, Jeonju, Republic of Korea; 3 KM Science Research Division, Korea Institute of Oriental Medicine, Daejeon, Republic of Korea; 4 CNS Pharm Korea Co., Ltd., Seoul, Republic of Korea

**Keywords:** flavonoid glucuronide, NF-κB, periodontitis, PI3K/AKT signaling, *Pimpinella brachycarpa*, network pharmacology, molecular docking

## Abstract

**Introduction:**

This study was conducted to investigate the preventive effects of *Pimpinella brachycarpa* extract (PBE), which has demonstrated anti-inflammatory effects but whose molecular mechanisms and active components are not completely understood, in experimental periodontitis and to elucidate its underlying mechanisms.

**Methods:**

The protective effects of PBE were evaluated in a lipopolysaccharide (LPS)-induced periodontitis rat model by assessing histological changes, TRAP-positive osteoclast presence, and alveolar bone loss. Network pharmacology, protein–protein interaction network analysis, KEGG pathway enrichment analysis, and molecular docking were performed to predict the potential targets and mechanisms of PBE. The anti-inflammatory effects of PBE were further investigated in RAW264.7 macrophages and human periodontal ligament fibroblasts (PDLFs). Inflammatory mediators, cytokines, matrix metalloproteinases (MMPs), and PI3K/Akt/NF-κB signaling-related proteins were analyzed. Major constituents of PBE were identified using UPLC-QTOF-MS, and their anti-inflammatory activities were evaluated *in vitro*.

**Results:**

PBE attenuated periodontal tissue destruction in LPS-induced periodontitis rats, as demonstrated by reduced inflammatory cell infiltration, decreased CEJ–ABC distance, and suppression of TRAP-positive osteoclast presence. Network pharmacology analysis identified the PI3K/Akt signaling pathway as a key target associated with the anti-inflammatory effects of PBE. In RAW264.7 macrophages, PBE significantly reduced the production of NO, PGE2, TNF-α, and IL-1β. In PDLFs, PBE suppressed IL-1β-induced production of PGE2, IL-6, and IL-1β and downregulated the expression of MMP-3, MMP-8, and MMP-13. Furthermore, PBE inhibited the phosphorylation of PI3K, Akt, IKK, IκBα, and NF-κB p65. Chemical profiling identified chlorogenic acid, luteolin 7-O-glucuronide, apigenin 7-O-glucuronide, and luteolin 3′-O-glucuronide as major constituents. Among these, flavonoid glucuronides exhibited stronger anti-inflammatory activities than chlorogenic acid *in vitro*.

**Conclusion:**

The present findings indicate that PBE exerts anti-inflammatory and tissue-protective effects in experimental models of periodontitis. The observed effects were associated with suppression of PI3K/Akt–NF-κB signaling. In addition, flavonoid glucuronides identified in PBE exhibited anti-inflammatory activities *in vitro*. Further studies are required to clarify the contribution of individual constituents to the biological activity of PBE.

## 1 Introduction

Periodontitis is a chronic inflammatory disease characterized by progressive tissue destruction. It is primarily triggered by specific Gram-negative anaerobic pathogens that activate macrophages and other immune cells to produce proinflammatory cytokines, including tumor necrosis factor-alpha (TNF-α), interleukin-1 beta (IL-1β), and prostaglandin E2 (PGE2), which further amplify the local inflammatory response (Kwon et al., 2021; Martinez-Garcia and Hernandez-Lemus, 2021). Subsequently, the excessive production of matrix metalloproteinases (MMPs) causes collagen fiber degradation and alveolar bone destruction. These pathological changes result in the clinical manifestations of periodontitis, including clinical attachment loss, increased probing depth, bleeding during probing, and tooth mobility (Kwon et al., 2021).

Periodontitis affects nearly half of adults worldwide and has significant impacts on oral and systemic health. Accumulating evidence indicates bidirectional links with systemic diseases such as diabetes, cardiovascular disease, and rheumatoid arthritis (Hu et al., 2025; Tonetti et al., 2017). The primary goals of periodontal treatment are to control infection, reduce inflammation, and prevent further tissue destruction. Recent research interest has shifted to host-modulating therapies that target the inflammatory response. Doxycycline administered at a subantimicrobial dose, nonsteroidal anti-inflammatory drugs, and various phytochemicals have been studied for their efficacy at doses lower than those of conventional antibiotics. However, limitations such as antibiotic resistance, incomplete regeneration, and recurrent inflammation remain. Therefore, there is a growing need for novel, safe, and multi-target anti-inflammatory agents, particularly from natural products (Li et al., 2024; Golub et al., 2024; Malcangi et al., 2025). Natural product extracts reportedly exhibit various clinical effects, including anti-inflammatory, antioxidant, and antimicrobial activities. Natural bioactive compounds have been proposed as alternatives to existing treatments (Song, 2024; Merecz-Sadowska et al., 2020).

*Pimpinella brachycarpa* (Kom.) Nakai, a perennial herb belonging to the Apiaceae family, is widely distributed in mountainous regions in Asia, Africa, and Europe. In Korea, it is consumed as a traditional edible plant as part of folk remedies for persistent cough, bronchial asthma, gastrointestinal disorders, and insomnia. It is also used as a culinary and functional ingredient (Ahn et al., 2011). In previous studies, various bioactive compounds, including terpenes and flavonoids, have been identified (Lee et al., 2013; Kim et al., 2020a). Several studies have reported the antioxidant, antifungal, antithrombotic, anticancer, hepatoprotective, and antibacterial properties of *P. brachycarpa* extract (PBE) (Kim et al., 2020b; Kwon et al., 2004). Our previous study demonstrated that PBE attenuated periodontal inflammation and alveolar bone destruction in a ligature-induced periodontitis rat model and suggested chlorogenic acid as one of the contributing components (Park et al., 2026). Although the anti-inflammatory efficacy of PBE has been demonstrated *in vivo*, the precise molecular mechanisms and target pathways underlying its beneficial effects remain unclear.

In this study, we aimed to investigate the protective effects and underlying mechanisms of PBE in lipopolysaccharide (LPS)-induced periodontitis. The specific objectives were: (i) to construct a compound-target gene network of PBE using network pharmacology analysis and predict the interactions between key active compounds and target proteins associated with periodontitis through molecular docking, and (ii) to experimentally validate the predicted mechanisms using RAW264.7 macrophages and human periodontal ligament fibroblasts (PDLFs) to evaluate the anti-inflammatory effects of PBE *in vitro*.

## 2 Materials and methods

### 2.1 Phytochemical analysis of PBE

*Pimpinella brachycarpa* (Kom.) Nakai samples were obtained from Gyeongdong Market (Seoul, Republic of Korea), and PBE was supplied by CNS Pharm Korea (Seoul, Republic of Korea). The study material was identified by Dr. Sungyu Yang at Korea Institute of Oriental Medicine (KIOM) and a voucher specimen (no. YSG_KIOM-2026-44) was deposited at the Korean Herbarium of Standard Herbal Resources at KIOM. Fresh samples were oven-dried at 45 °C and extracted with 50% ethanol at reflux. The extract was concentrated, spray-dried, and stored at <4 °C. Phytochemical analysis was conducted using high-performance liquid chromatography (HPLC). Detailed methods are presented in Supplementary Material S1.

### 2.2 Experimental design and treatment evaluation in an LPS-induced rat periodontitis model

A periodontitis model was established by locally injecting *Porphyromonas gingivalis*-derived LPS into the gingiva surrounding the maxillary first molars of 6-week-old male Sprague–Dawley rats (n = 5 per group). LPS (1 mg/mL, 1 µL) was injected into the lingual gingiva with the rats under ketamine/xylazine anesthesia, and a second injection was administered 3 days later. Control animals received an equivalent volume of saline. Test substances were orally administered once daily for 14 days starting after the initial LPS administration. Insadol® plus (ISD; Dongkook Pharmaceutical Co., Ltd., Seoul, Republic of Korea; 100 mg/kg/day) was used as a positive control. PBE was administered at doses of 37.5, 75, and 150 mg/kg/day based on the results of our previous studies (Park et al., 2026). The rats were euthanized and maxillae were collected for analysis on day 15. Histological evaluation was performed on decalcified paraffin sections stained with hematoxylin and eosin (H&E) to assess periodontal inflammation, alveolar bone loss (measured as the cementoenamel junction (CEJ)–alveolar bone crest (ABC) distance), and root resorption. The degree of periodontal inflammation was assessed using a histological scoring system (Table 1) ranging from 0 (absence) to 4 (severe), based on inflammatory cell infiltration, epithelial disruption, and ABC changes. Immunohistochemical staining for CD45 and tartrate-resistant acid phosphatase (TRAP) was conducted to evaluate inflammatory cell infiltration and the presence of TRAP-positive osteoclasts, respectively. Quantitative analyses were performed using ImageJ software under blinded conditions. All procedures were approved by the Institutional Animal Care and Use Committee of Jeonbuk National University Hospital (Approval No. JBNUH-IACUC-2025-22). Detailed methods are presented in Supplementary Material S2.

**TABLE 1:** Inflammation scoring system.

Score	Degree	Lesions
0	Absence	None
1	Slight	Gingivitis: Slight hyperplasia of gingival epithelium (GE) and intraepithelial inflammatory cell infiltration. Accumulation of bacterial plaque. No substantial changes in the lamina propria (LP), periodontal ligament (PDL), or alveolar bone crest (ABC)
2	Mild	Mild gingival hyperplasia, inflammatory cell infiltration of the GE and LP, accumulation of bacterial plaque, and slight disruption of the PDL. No substantial change in ABC.
3	Moderate	Erosion/ulceration and hyperplasia of the GE and accumulation of bacterial plaque. Moderate inflammatory cell infiltration of the LP and disruption of the PDL. Migration of the junctional epithelium and ABC resorption
4	Severe	Ulceration and hyperplasia of the GE. Severe inflammatory cell infiltration of the LP, disruption of the PDL, migration of the junctional epithelium, and severe ABC resorption and osteolysis

### 2.3 *In vitro* evaluation of anti-inflammatory effects in RAW264.7 macrophages and human PDLFs

RAW264.7 murine macrophages and human PDLFs were cultured in Dulbecco’s modified Eagle’s medium supplemented with 10% fetal bovine serum and 1% penicillin/streptomycin at 37 °C in a humidified atmosphere containing 5% CO_2_. Cells were seeded and pre-treated with various concentrations of PBE (50, 100, and 200 μg/mL) or its constituent compounds (apigenin 7-glucuronide [AG], luteolin 7-glucuronide [LG], and chlorogenic acid [CA]; 5–100 μg/mL) for 2 h, followed by stimulation with LPS or IL-1β for 24 h. Cell viability was assessed using the Cell Counting Kit-8 assay. Nitric oxide production was measured using Griess reagent and inflammatory mediators, including TNF-α, IL-1β, IL-6, PGE2, and MMP-3 were quantified using enzyme-linked immunosorbent assay. Protein expression was analyzed using Western blotting, with β-actin used as an internal control. Expression levels of *MMP-3*, *MMP-8*, and *MMP-13* were determined using quantitative reverse transcription polymerase chain reaction, normalized to *GAPDH* expression, and calculated using the 2^−ΔΔCt^ method. Detailed methods are presented in Supplementary Material S3.

### 2.4 Network pharmacology analysis and molecular docking

The four compounds used for network pharmacology and molecular docking analyses were selected based on HPLC identification using authentic reference standards. As these compounds represented the major chemically characterized constituents detected in PBE, they were chosen as candidate bioactive compounds for subsequent *in silico* analyses. Information on the four compounds (CA, AG, LG, and luteolin 3′-glucuronide) was collected from the PubChem database. Potential target genes of each compound were predicted using multiple databases, including STITCH, BATMAN-TCM, SwissTargetPrediction, TM-MC, SEA, and PubChem. Periodontal disease-related genes were obtained from the GeneCards database using the keyword “periodontal disease”. Overlapping genes between compound targets and disease-related human gene were identified as potential targets and visualized using a Venn diagram.

Protein–protein interaction (PPI) analysis of the potential targets was conducted using the STRING database and visualized in Cytoscape. Pathway enrichment analysis was performed using STRING, DAVID, and Kyoto Encyclopedia of Genes and Genomes (KEGG) databases, and the results were visualized as bubble plots based on significance and gene counts.

Molecular docking analysis was performed between the active compounds and key inflammatory targets (IL1B, IL6, IL8, iNOS, MMP3, PTGS2, and TNFA). Protein structures were obtained from the Protein Data Bank and docking simulations were conducted using AutoDock Vina. Binding affinities were evaluated based on predicted binding energy values. Detailed methods are presented in Supplementary Material S4.

### 2.5 Statistical analysis

Statistical analyses were performed using GraphPad Prism (version 7.0; GraphPad Software Inc., San Diego, CA, USA). For multiple comparisons, one-way analysis of variance with Dunnett’s *post hoc* test (parametric) was used. Statistical significance was set at *p* < 0.05 for all experiments.

## 3 Results

### 3.1 Quantification of marker compounds of PBE

We identified four primary phytochemicals present in PBE by comparing the retention time and mass spectra with reference standards using HPLC. The quantitative analysis results were as follows: 22.7 ± 0.03 mg/g chlorogenic acid, 7.5 ± 0.08 mg/g luteolin 7-O-glucuronide, 3.3 ± 0.01 mg/g apigenin 7-O-glucuronide, and 4.9 ± 0.03 mg/g luteolin 3′-O-glucuronide (Figure 1).

**FIGURE 1:**
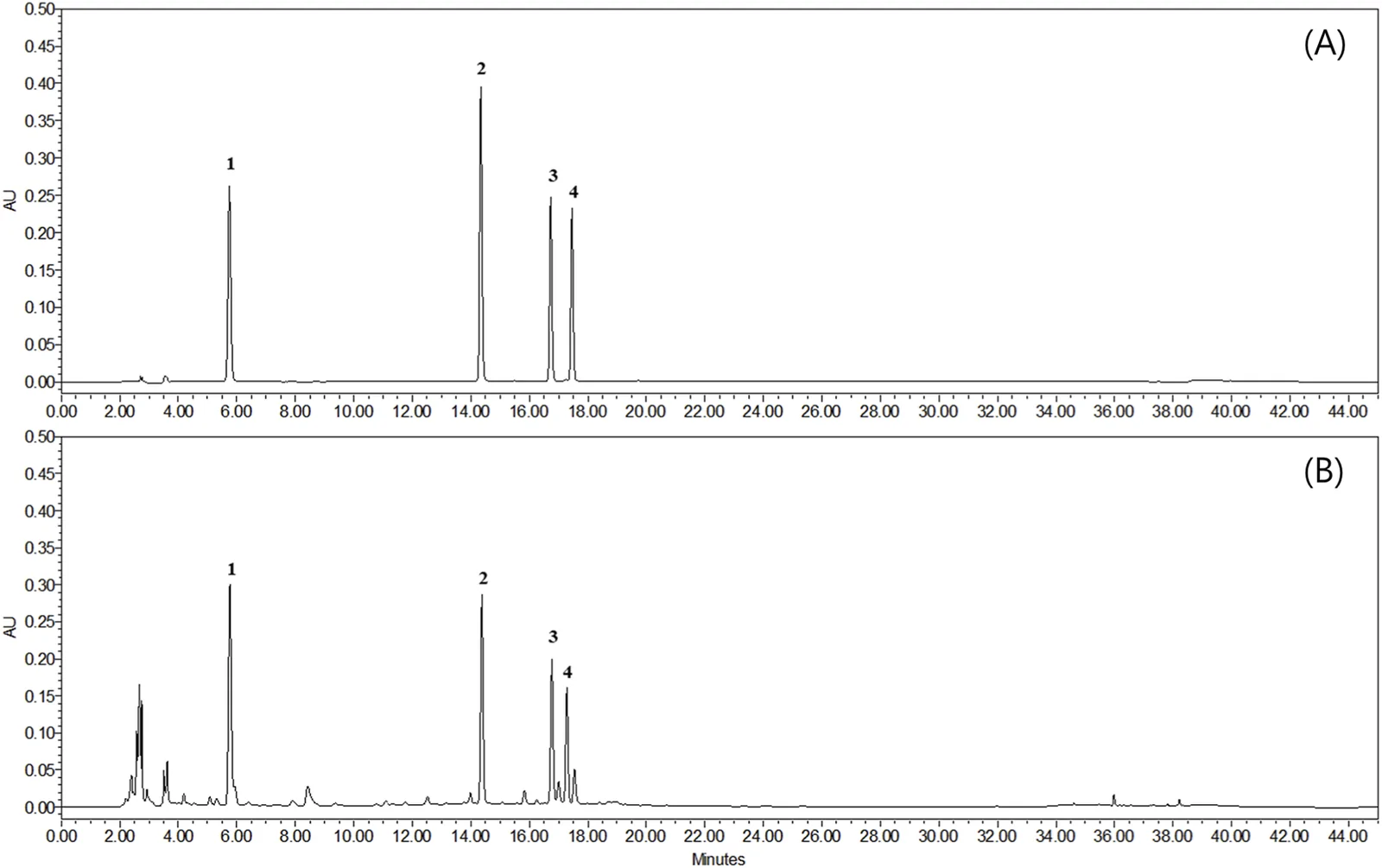
High-performance liquid chromatography (HPLC) chromatogram of *Pimpinella brachycarpa* extract (PBE) at 250 nm. **(A)** Reference standard mixture. **(B)** PBE. Major components were identified as (1) chlorogenic acid, (2) luteolin 7-O-glucuronide, (3) apigenin 7-O-glucuronide, and (4) luteolin 3′-O-glucuronide.

### 3.2 PBE attenuates LPS-induced alveolar bone loss and reduces superficial root resorption as determined using histological analysis

H&E staining was used to assess alveolar bone loss and superficial root resorption in LPS-induced periodontitis. The CEJ–ABC distance was significantly greater in the LPS group than that in the control group (Figure 2A). Conversely, ISD and all PBE-treated groups showed a significant reduction in this distance, demonstrating that PBE attenuated LPS-induced alveolar bone loss. Additionally, the LPS group showed a marked increase in resorption lacunae along the root surface compared with the control group (Figure 2C), whereas the number of resorption lacunae tended to decrease in the ISD and PBE groups, suggesting a protective effect.

**FIGURE 2:**
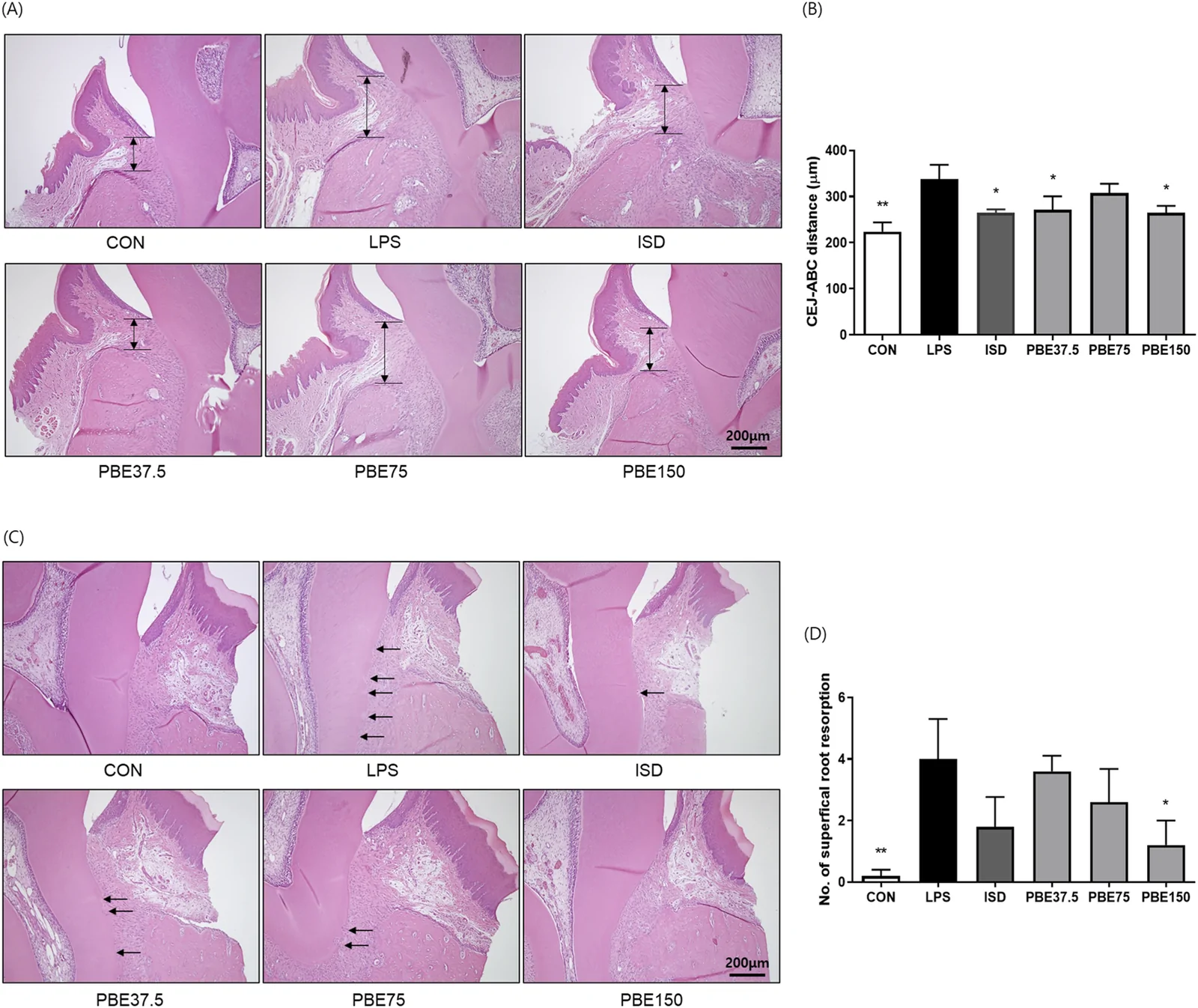
Histological assessment of the protective effects of *Pimpinella brachycarpa* extract (PBE) on alveolar bone loss and superficial root resorption in lipopolysaccharide (LPS)-induced periodontitis. **(A)** Hematoxylin and eosin (H&E)-stained sections of the LPS group showed substantial bone loss and structural collapse, which were alleviated following ISD and PBE treatment. **(B)** The cementoenamel junction (CEJ)-to-alveolar bone crest (ABC) distance was significantly reduced in rats treated with PBE, indicating preservation of periodontal structure. **(C)** Representative H&E-stained sections illustrating superficial root resorption in each experimental group. **(D)** Quantitative analysis of superficial root resorption. Data are presented as mean ± standard deviation (SD). *p < 0.05, **p < 0.01 vs. LPS.

### 3.3 PBE alleviates LPS-induced periodontal inflammation and reduces CD45-positive inflammatory cell infiltration

Periodontitis severity was evaluated using a histological scoring system (Figure 3A). The LPS group showed a higher periodontitis score than that of the control group, indicating successful induction of inflammatory changes. The periodontitis score of the ISD and PBE groups exhibited a decreasing trend, with the high-dose PBE (150 mg/kg) group showing a significant reduction. CD45 immunohistochemical staining was performed to evaluate the infiltration of inflammatory cells in periodontal tissues (Figure 3B). The LPS group showed a marked increase in the proportion of CD45- positive cells compared with that of the control group, indicating enhanced inflammatory cell recruitment. Although the ISD and PBE groups did not show significant differences, both groups exhibited a tendency toward a decrease in the CD45-positive staining area compared with that of the LPS group (Figure 3C).

**FIGURE 3:**
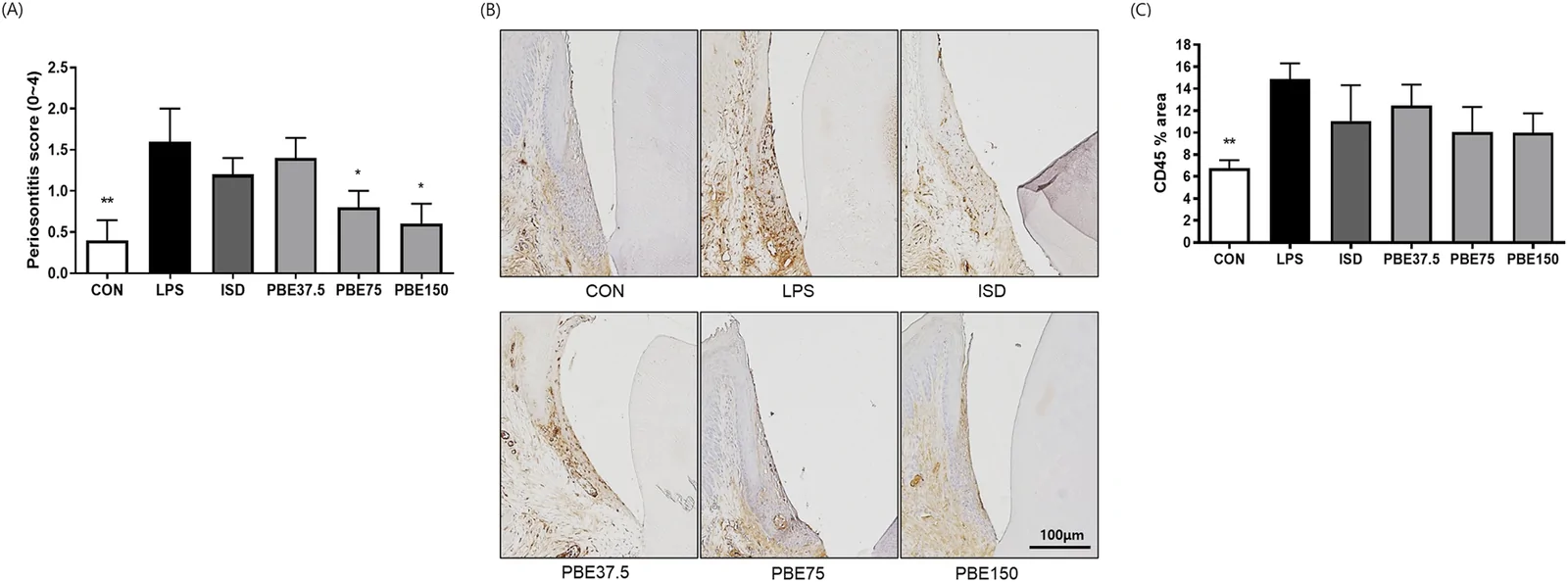
Effects of PBE on periodontal inflammation and inflammatory cell infiltration in lipopolysaccharide (LPS)-induced periodontitis. **(A)** Inflammation scoring was performed according to the extent of inflammatory cell infiltration, epithelial disruption, and alveolar bone crest (ABC) changes from 0 (absence) to 4 (severe). **(B)** Immunohistochemical staining showed strong CD45 signals in the LPS group, reflecting marked inflammation. **(C)** Quantitative analysis demonstrated that PBE tended to reduce the CD45-positive staining area, supporting its potential anti-inflammatory effects. Data are expressed as mean ± standard deviation (SD). *p < 0.05, **p < 0.01 vs. LPS.

### 3.4 PBE suppresses TRAP-positive osteoclast presence in LPS-induced periodontitis

TRAP staining was performed to evaluate the presence of TRAP-positive osteoclasts in periodontal tissues (Figure 4A). The LPS group had a markedly higher number of TRAP-positive multinucleated cells than that of the control group. In contrast, the ISD and PBE groups exhibited a reduction in TRAP-positive areas, with a significant decrease observed in the PBE75 and PBE150 groups (Figure 4B). These findings suggest that PBE suppresses TRAP-positive osteoclast accumulation in LPS-induced periodontal tissues.

**FIGURE 4:**
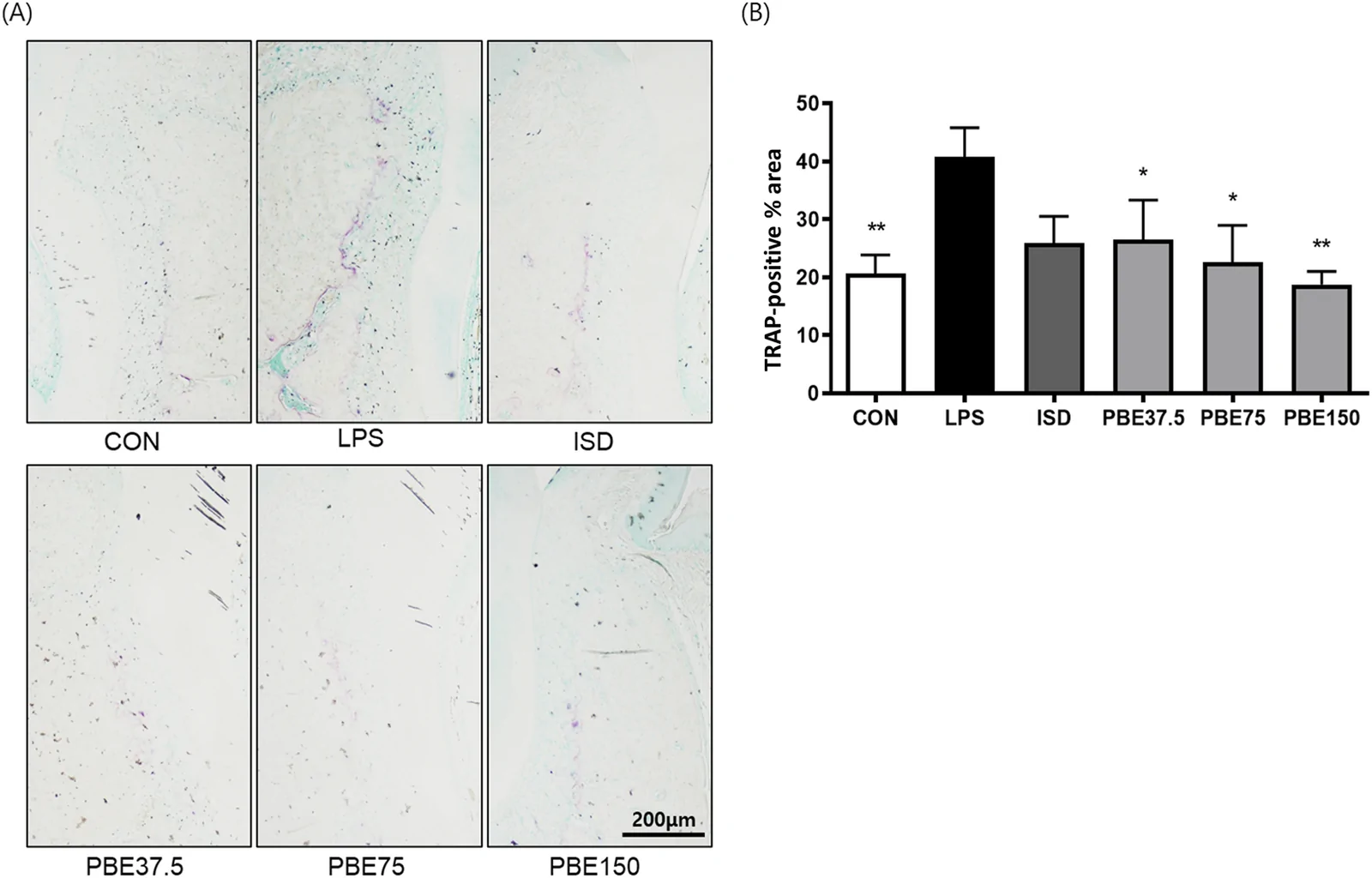
Effect of *Pimpinella brachycarpa* extract (PBE) on TRAP-positive osteoclast presence in lipopolysaccharide (LPS)-induced periodontitis. **(A)** Tartrate-resistant acid phosphatase (TRAP) staining demonstrated a marked increase in osteoclast proportion in the LPS group, whereas treatment with PBE significantly reduced the number and staining intensity of TRAP-positive cells. **(B)** Quantitative analysis of TRAP-positive area demonstrated that PBE significantly suppressed TRAP-positive area in a dose-dependent manner. *p < 0.05, **p < 0.01 vs. LPS.

### 3.5 Analysis of compound–target gene and PPI network

The four compounds were associated with 414 predicted target genes, of which 184 overlapped with 3,442 periodontal disease-related genes identified through intersection analysis (Figure 5A). It should be noted that the periodontal disease-related genes retrieved from GeneCards were not restricted to a single pathological phenotype. Instead, they encompassed multiple biological processes involved in periodontal disease progression, including inflammatory responses, extracellular matrix degradation, fibroblast dysfunction, and bone remodeling. The multi-set Venn diagram analysis revealed that 14 proteins were commonly associated with all 4 compounds among the 184 proteins overlapping with periodontal disease-related targets (Figures 5B,C).

**FIGURE 5:**
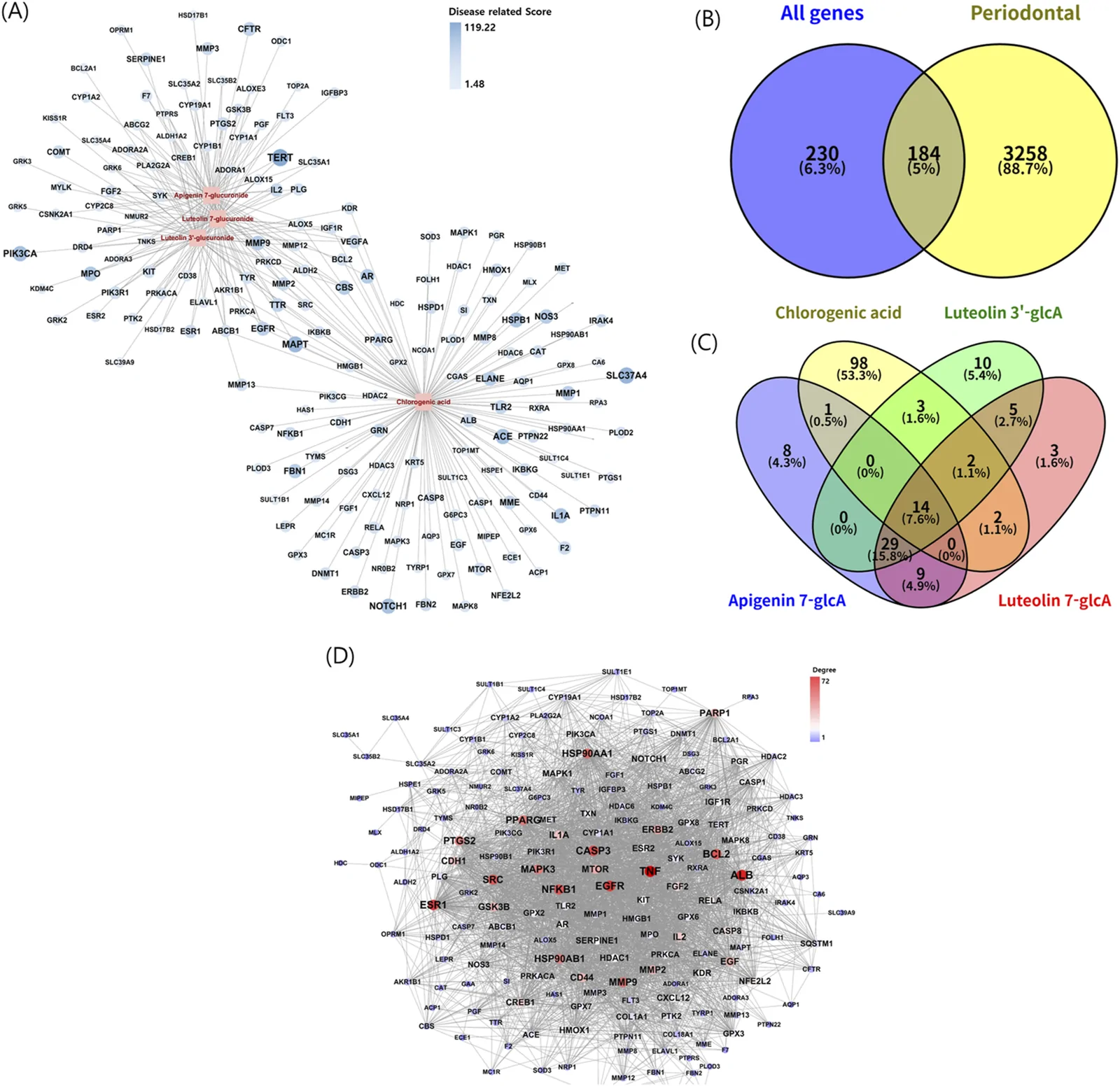
Network pharmacology analysis of *Pimpinella brachycarpa* extract (PBE) for periodontal disease. **(A)** Compound–target interaction network of four flavonoid-derived compounds (apigenin 7-glucuronide, luteolin 3′-glucuronide, luteolin 7-glucuronide, and chlorogenic acid). **(B)** Venn diagram illustrating the overlap between predicted compound-associated targets and periodontal disease-related genes. **(C)** Venn diagram showing shared and unique target genes among the four compounds: apigenin 7-glucuronide (blue), chlorogenic acid (yellow), luteolin 3′-glucuronide (green), and luteolin 7-glucuronide (red), highlighting common and compound-specific multi-target interaction profiles. **(D)** Protein–protein interaction (PPI) network constructed from the overlapping target genes associated with the tested flavonoid-derived compounds and inflammation-related disease genes.

To evaluate the topological properties of the PPI network, hub genes were ranked based on degree. TNF, ALB, EGFR, ESR1, CASP3, NFKB1, BCL2, SRC, HSP90AA1, and MMP9 were consistently ranked among the top nodes, indicating their structurally central positions within the network (Figure 5D).

### 3.6 Enrichment analysis of PBE-associated targets in periodontal disease

Gene Ontology enrichment analysis revealed that PBE-associated target genes were primarily localized to extracellular and cell surface-associated compartments and were functionally involved in key pathological processes of periodontitis, including inflammation, regulation of apoptosis, oxidative stress responses, and extracellular matrix (ECM) disassembly. The KEGG pathway enrichment analysis focusing on periodontal disease-related pathways revealed significant enrichment of major inflammatory and immune signaling pathways, such as the PI3K-Akt, MAPK, NF-κB, TNF, IL-17, and Toll-like receptor signaling pathways. The pathway–gene network analysis demonstrated substantial overlap among these pathways, identifying a few hub genes, such as IKBKB, IKBKG, MAPK1, MAPK3, NFKB1, and RELA, recurrently involved in multiple periodontal disease-related signaling pathways (Figure 6). Collectively, these results suggest that PBE exerts its effects by targeting key extracellular and cell surface-associated inflammatory signaling networks through central hub genes in periodontitis.

**FIGURE 6:**
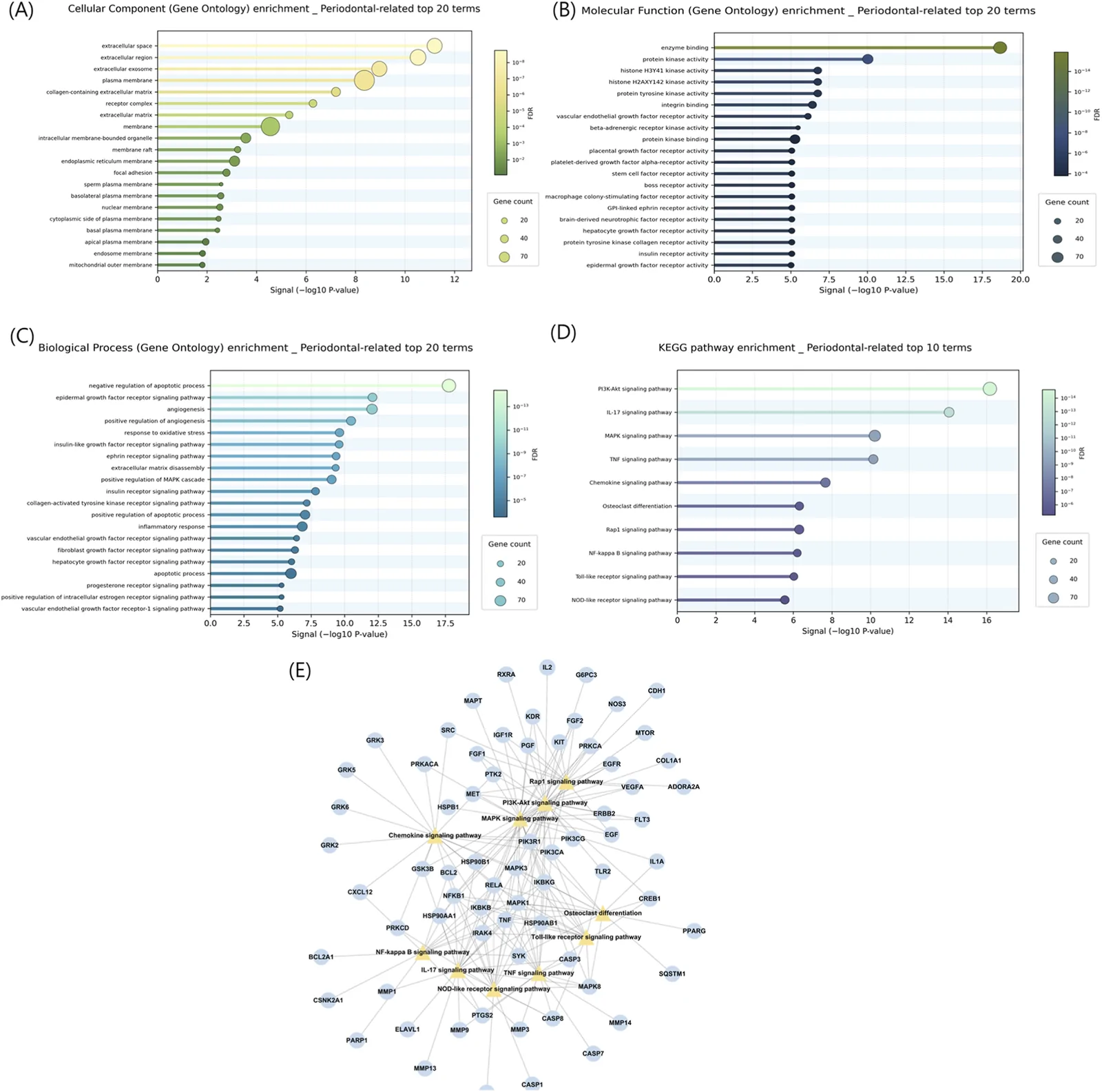
Enrichment analyses of the predicted target genes. Gene Ontology (GO) enrichment analysis of periodontal disease-related target genes; **(A)** cellular component (CC), **(B)** molecular function (MF), and **(C)** biological process (BP) enrichment analysis of the top 20 GO terms. In all panels, the x-axis represents enrichment significance expressed as −log10(P-value). Dot size indicates the number of genes associated with each GO term and the color intensity reflects the false discovery rate (FDR). **(D)** Kyoto Encyclopedia of Genes and Genomes (KEGG) pathway enrichment analysis of periodontal disease-associated target genes. The top 10 significantly enriched pathways are shown, ranked by −log10(P-value). Dot size represents the number of genes involved in each pathway and color indicates the FDR. **(E)** Pathway–gene interaction network illustrating relationships between the enriched KEGG pathways and associated target genes.

### 3.7 Molecular docking

Molecular docking was used to assess the predicted binding affinities of the four flavonoid-derived compounds—AG, CA, luteolin 3′-glucuronide, and LG—toward seven inflammation-related targets—IL1B, IL6, IL8, iNOS, MMP3, PTGS2, and TNFA. The results revealed clear target- and compound-dependent binding patterns. iNOS and PTGS2 showed the strongest predicted binding with all four compounds, with LG exhibiting the highest affinities, followed by AG. Flavonoid glucuronides also displayed relatively strong binding to TNFA and moderate binding to MMP3, whereas CA generally showed weaker affinities. Furthermore, AG and LG demonstrated slightly stronger binding than CA to IL1B, IL6, and IL8 (Figure 7). Overall, these findings suggest that flavonoid glucuronides, particularly LG and AG, possess a more robust multi-target anti-inflammatory binding profile than CA. To further evaluate the functional relevance of these interactions, docking poses were examined within the known active or substrate-recognition regions of iNOS, PTGS2, and MMP3. The compounds were localized within the catalytic or functional binding pockets and interacted with key active-site residues, including His201, His205, and His211 in MMP3, Arg120, Tyr355, and Ser530 in PTGS2, and residues lining the catalytic pocket of iNOS. Representative binding poses and residue-level interaction analyses are presented in Figure 7.

**FIGURE 7:**
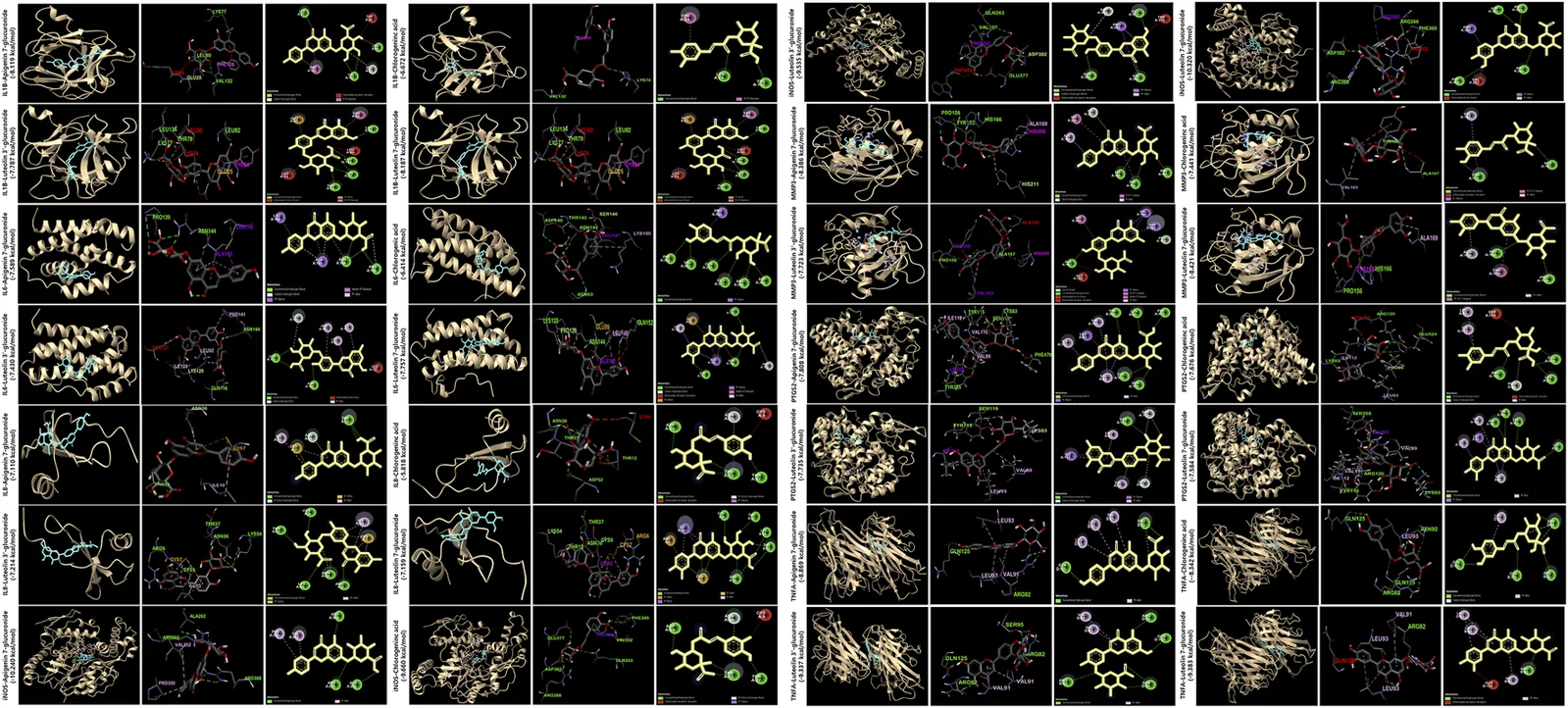
Molecular docking analysis of four representative compounds with inflammatory target proteins.

### 3.8 Regulatory effects of PBE on the secretion of inflammatory mediators and cytokines in RAW264.7 macrophages and PDLFs

To investigate the anti-inflammatory effects of PBE, we examined pro-inflammatory markers in macrophages and PDLFs. No significant reduction in cell viability was observed up to 200 μg/mL, which was the highest concentration tested in this study. LPS significantly upregulated the levels of inflammatory mediators and cytokines in RAW264.7 cells, whereas PBE treatment significantly decreased nitric oxide (NO), PGE2, TNF-α, and IL-1β secretion (Figures 8A–E). Furthermore, we determined the expression of inflammatory markers in IL-1β–stimulated PDLFs. In the IL-1β–stimulated group, the expression levels of IL-1β, IL-6, and PGE2 were significantly upregulated, whereas PBE suppressed their levels in a dose-dependent manner, particularly in the PBE group treated with 200 μg/mL PBE (Figures 8F–I). These results confirm that PBE exerts potent anti-inflammatory activity by attenuating cytokine production in macrophages and PDLFs.

**FIGURE 8:**
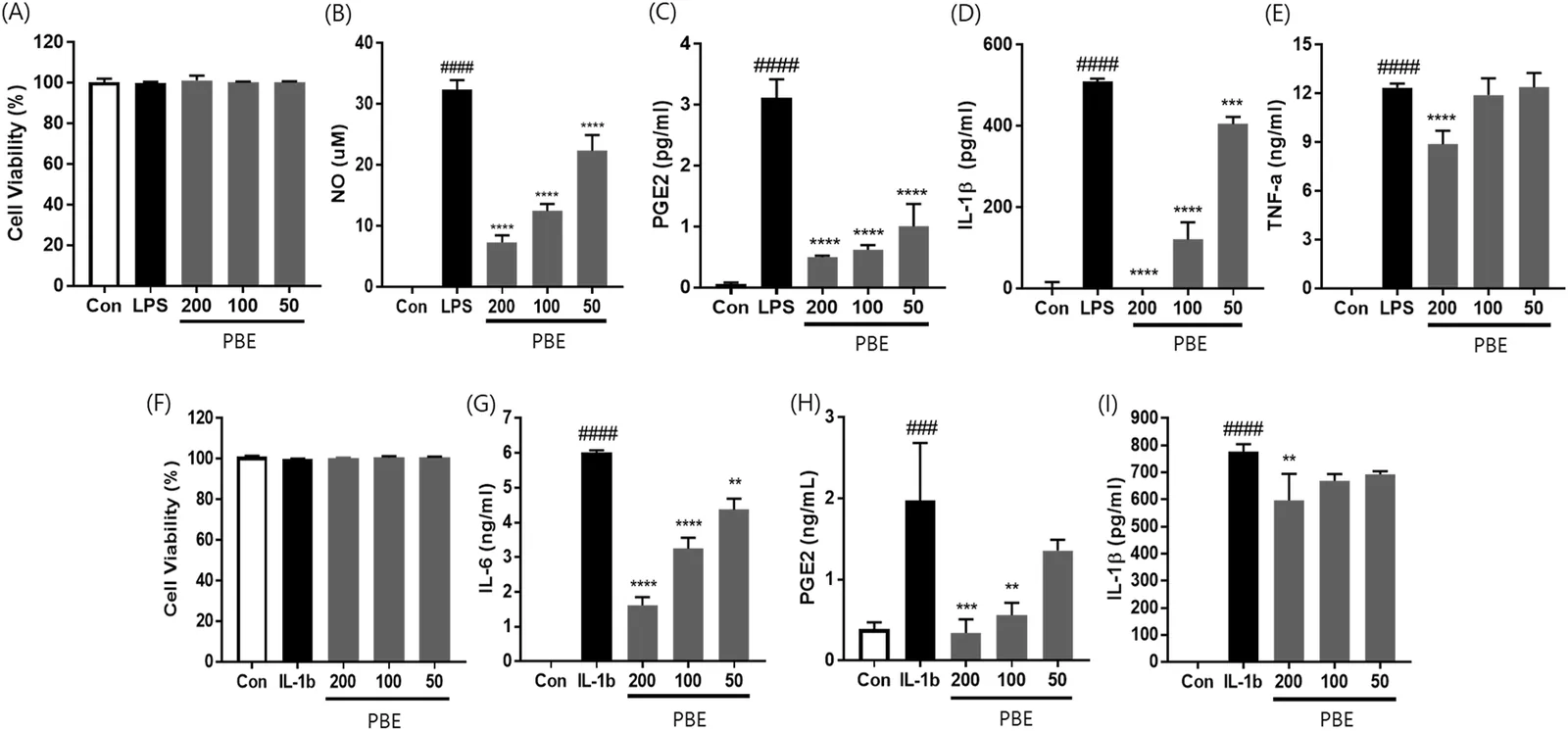
Effects of *Pimpinella brachycarpa* extract (PBE) on pro-inflammatory mediator and cytokine expression in RAW264.7 and periodontal ligament fibroblasts (PDLFs). **(A)** Effect of PBE on cell viability in the presence of lipopolysaccharide (LPS). Concentration of nitric oxide (NO) **(B)**, prostaglandin E_2_ (PGE_2_) **(C)**, interleukin-1 beta (IL-1β) **(D)**, and tumor necrosis factor-alpha (TNF-α) **(E)** produced in LPS-induced RAW264.7 cells. Effect of PBE on cell viability in the presence of IL-1β **(F)**. Concentration of IL-6 **(G)**, PGE2 **(H)**, and IL-1β **(I)** produced in IL-1β-exposed PDLFs. Statistical significance was determined by comparing the LPS- or IL-1β-treated groups with the control group (###*p* < 0.001, ####*p* < 0.0001). Additionally, differences between each sample and the LPS- or IL-1β-induced groups were evaluated (**p < 0.01, ***p < 0.001, ****p < 0.0001).

### 3.9 PBE inhibits ECM degradation in IL-1β–stimulated PDLFs

To evaluate the effect of PBE on MMP expression, we assessed the mRNA levels of *MMP-3, MMP-8,* and *MMP-13* in PDLFs stimulated with IL-1β. The expression of *MMP-3, MMP-8, and MMP-13* was markedly higher in the IL-1β–stimulated group than that in the control group. However, pre-treatment with PBE resulted in a dose-dependent reduction in MMP expression. Notably, *MMP-3, MMP-8,* and *MMP-13* expression in the 200 μg/mL PBE group was significantly lower than that in the IL-1β–treated group. These results suggest that PBE attenuates inflammatory stimulation-induced ECM degradation in PDLFs by downregulating the expression of key MMPs, which may contribute to the reduction of periodontal tissue destruction (Figure 9).

**FIGURE 9:**
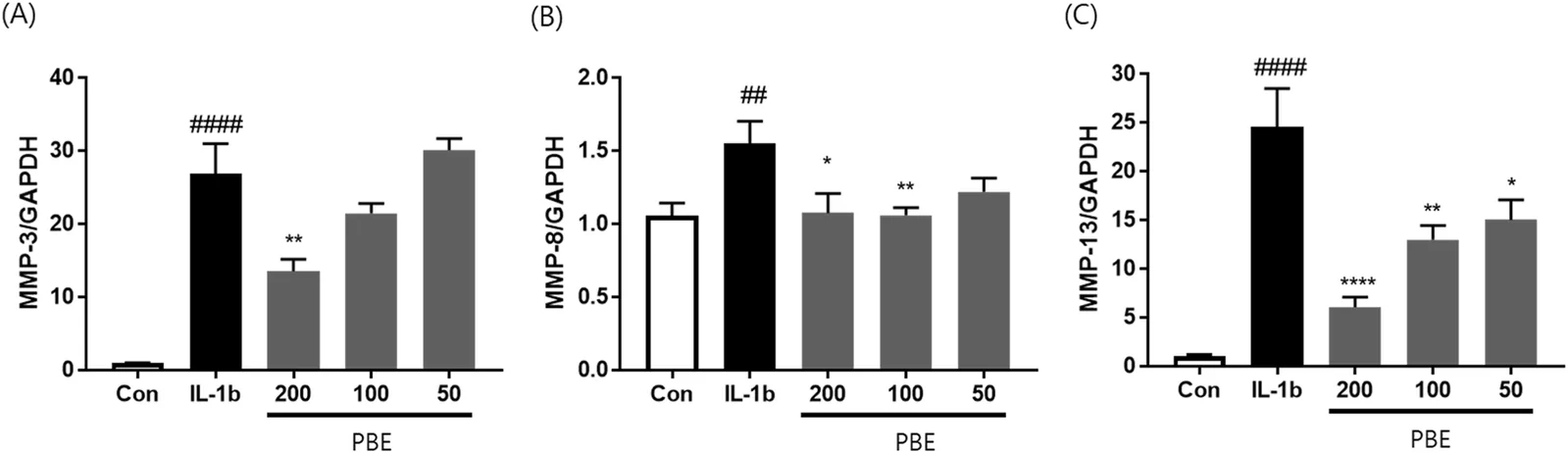
Effects of *Pimpinella brachycarpa* extract (PBE) on mRNA expression levels of matrix metalloproteinase (MMP)-3 **(A)**, –8 **(B)**, and –13 **(C)** in interleukin-1 beta (IL-1β)–stimulated periodontal ligament fibroblasts (PDLFs). Values in the bar graphs are presented as mean ± standard deviation (SD). ##*p* < 0.01, ####*p* < 0.0001 vs. the control group (Con); *p < 0.05, **p < 0.01, ****p < 0.0001 vs. the IL-1β group.

### 3.10 PBE is associated with reduced IL-1β–induced activation of the PI3K/AKT and NF-κB signaling pathway in PDLFs

To clarify the anti-inflammatory mechanism of PBE, we evaluated its effects on the PI3K/Akt and NF-κB signaling pathways in IL-1β–stimulated PDLFs. IL-1β markedly increased PI3K and AKT phosphorylation, whereas PBE pre-treatment significantly suppressed these changes in a dose-dependent manner. Simultaneously, IL-1β activated the NF-κB pathway by increasing IKKα/β, IκBα, and p65 phosphorylation and decreasing total IκBα expression. PBE inhibited these IL-1β–induced alterations, reducing IKKα/β, IκBα, and p65 phosphorylation while preventing IκBα degradation. Collectively, these findings indicate that PBE attenuates inflammation in PDLFs through suppression of PI3K/Akt and NF-κB pathways (Figure 10).

**FIGURE 10:**
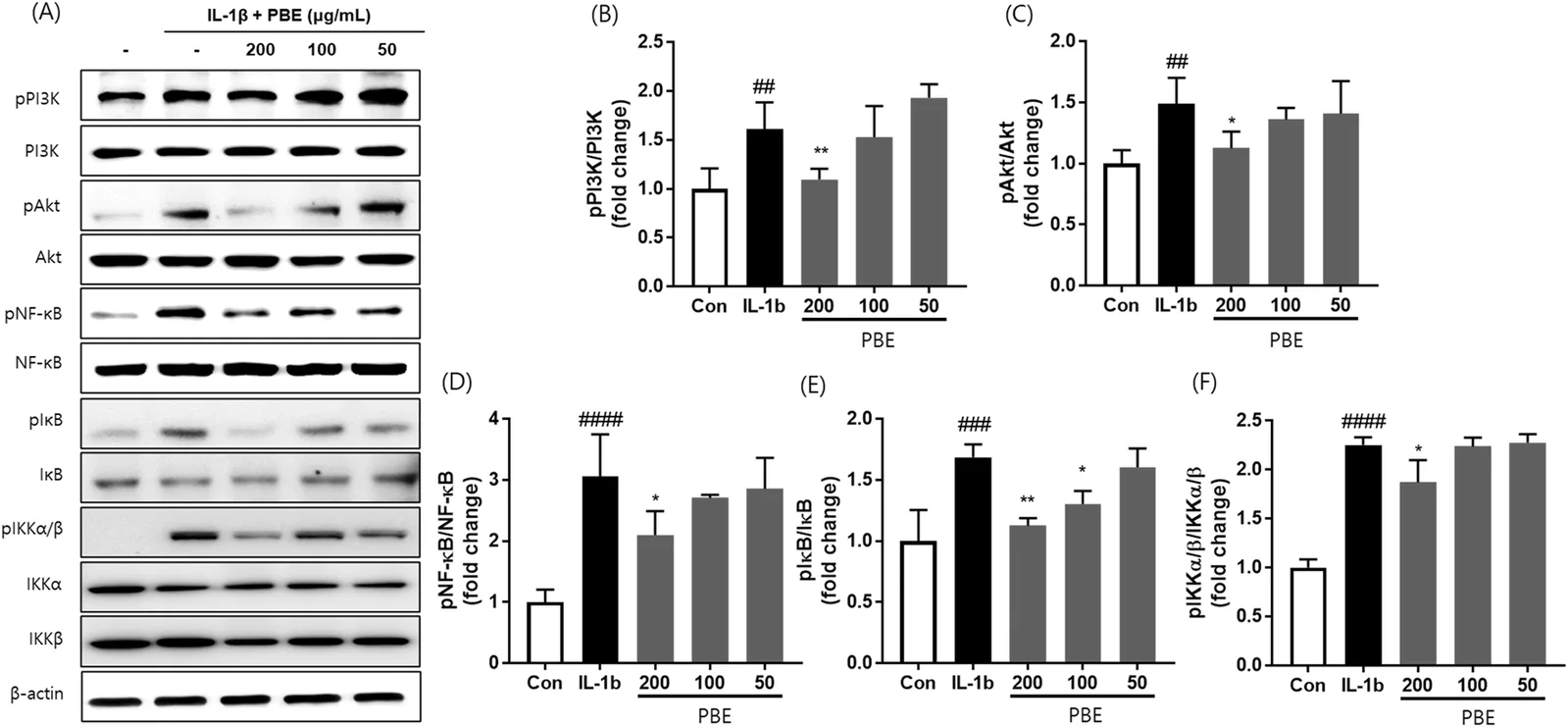
*Pimpinella brachycarpa* extract (PBE) inhibits interleukin-1 beta (IL-1β)–induced activation of the phosphatidylinositol 3-kinase/protein kinase B (PI3K/Akt) and nuclear factor-κB (NF-κB) signaling pathways. **(A)** Western blotting showing protein expression, p-PI3K/PI3K levels **(B)**, p-AKT/AKT levels **(C)**, p-p65/P65 levels **(D)**, p-IκB/IκB levels **(E)**, and p-IKKα/β/IKKα/β levels **(F)**. ##*p* < 0.01, ###*p* < 0.001, ####*p* < 0.0001 vs. the control group (Con); *p < 0.05, **p < 0.01 vs. the IL-1β group.

### 3.11 Regulatory effects of PBE-derived compounds on the secretion of inflammatory mediators and cytokines in RAW264.7 macrophages and PDLFs

To validate the molecular docking results, we compared the anti-inflammatory effects of AG, LG, and CA in macrophages and PDLFs. AG and LG significantly reduced the LPS-induced production of NO, PGE2, TNF-α, and IL-1β in macrophages in a dose-dependent manner, whereas CA exhibited weak effects even at high concentrations (Figures 11A–F). Similarly, in IL-1β–treated PDLFs, AG and LG showed stronger effects than CA (Figures 11G–J). Notably, AG exerted the most potent inhibitory effects on production of inflammatory mediators and cytokines in both cell types, demonstrating the strongest and most consistent anti-inflammatory activity across both cell systems.

**FIGURE 11:**
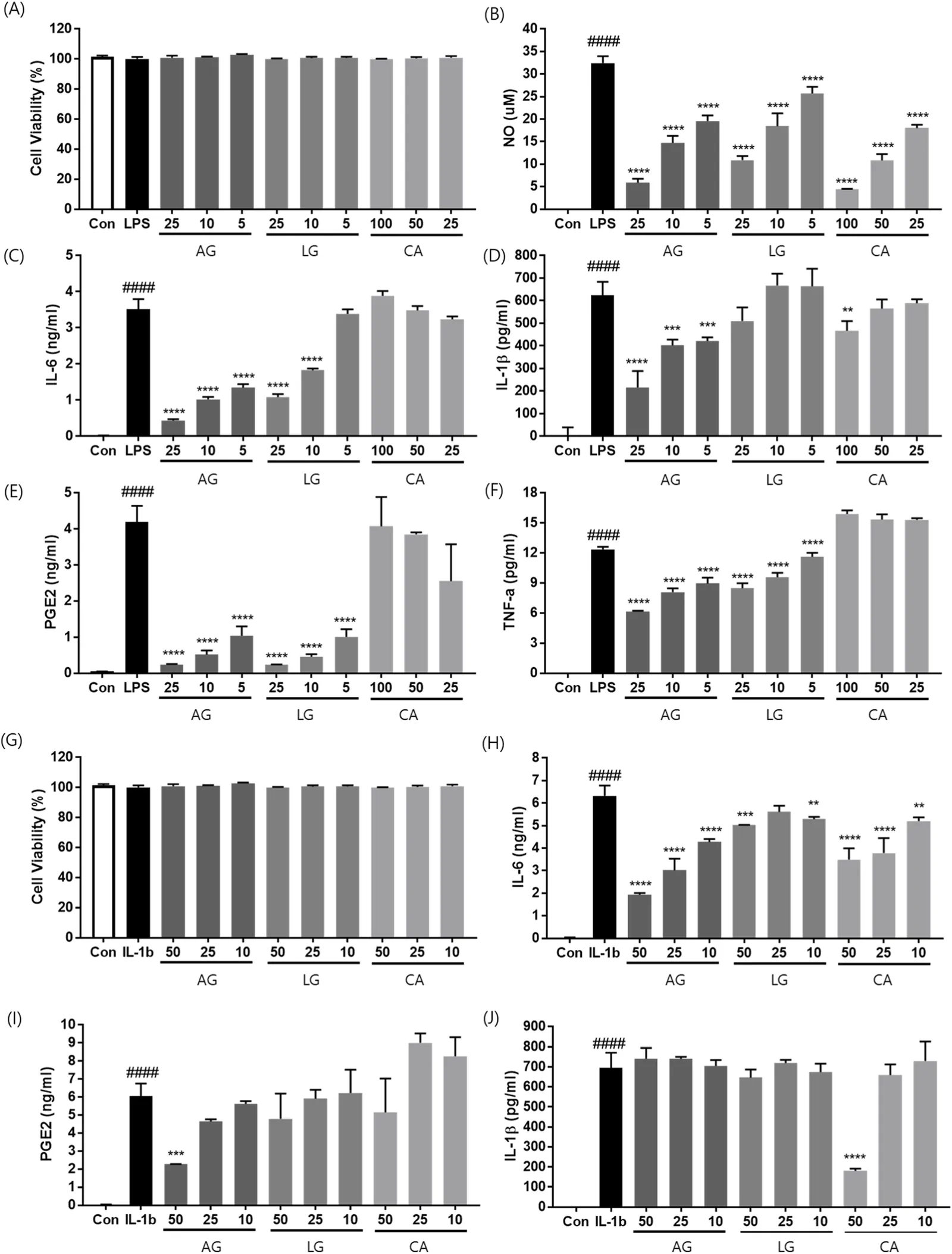
Expression of pro-inflammation mediators and cytokines in RAW264.7 and periodontal ligament fibroblasts (PDLFs). **(A)** Cell viability in the lipopolysaccharide (LPS)-induced RAW264.7 cells. Concentrations of nitric oxide (NO) **(B)**, interleukin-6 (IL-6) **(C)**, interleukin-1 beta (IL-1β) **(D)**, prostaglandin E_2_ (PGE_2_) **(E)**, and tumor necrosis factor-alpha (TNF-α) **(F)** in LPS-stimulated RAW264.7 cells pretreated with apigenin-7-glucuronide (AG), luteolin-7-glucuronide (LG), or chlorogenic acid (CA). **(G)** Viability of IL-1β-exposed PDLFs. Concentrations of IL-6 **(H)**, PGE_2_ **(I)**, and IL-1β **(J)** produced in IL-1β-exposed PDLFs. Statistical significance was determined by comparing the LPS- or IL-1β-treated groups with the control group (####*p* < 0.0001). Additionally, differences between each sample and the LPS- or IL-1β-induced groups were evaluated (**p < 0.01, ***p < 0.001, ****p < 0.0001).

## 4 Discussion

In this study, we evaluated the effects of PBE on periodontal inflammation and histological indicators of alveolar bone loss in an LPS-induced periodontitis model. LPS induces pro-inflammatory cytokines such as TNF-α, IL-1β, and IL-6, and enhances the expression of RANKL in periodontal tissues, thereby promoting osteoclast activation and alveolar bone destruction (Kim et al., 2020b; Park et al., 2026). Our *in vivo* findings suggest that PBE exerts anti-inflammatory effects and reduces histological indicators of periodontal tissue destruction in LPS-induced periodontitis, as evidenced by decreased inflammatory cell infiltration, reduced CEJ–ABC distance, and suppression of TRAP-positive osteoclast presence. These findings were consistent with those observed in our previous ligature-induced periodontitis model and provide further evidence supporting the beneficial effects of PBE in experimental periodontal inflammation (Park et al., 2026).

Network pharmacology was used to investigate the potential targets and pathways affected by PBE in periodontitis treatment. The PPI network analysis revealed ten major targets—TNF, ALB, EGFR, ESR1, CASP3, NFKB1, BCL2, SRC, HSP90AA1, and MMP9—potentially mediating the anti-inflammatory effects of PBE in periodontitis. Many of these targets are involved in the TNF–NF-κB axis, forming a central inflammatory network that may explain the effects of PBE (Alim et al., 2024). Recent evidence suggests that periodontitis is not regulated by a single signaling pathway but rather by a complex inflammatory network involving multiple interconnected pathways. Toll-like receptor-mediated recognition of periodontal pathogens activates several downstream signaling cascades, including PI3K/Akt, MAPK, NF-κB, and TNF-related pathways, which collectively regulate the production of inflammatory cytokines and tissue-destructive mediators. In addition, IL-17 signaling contributes to the amplification of periodontal inflammation by promoting cytokine production and matrix degradation. Consistent with these observations, our KEGG enrichment analysis identified PI3K-Akt, MAPK, TNF, IL-17, Toll-like receptor, and NF-κB signaling pathways as significantly enriched pathways associated with PBE-related targets in periodontitis (de Molon et al., 2026). Among these enriched pathways, the PI3K/Akt–NF-κB signaling axis was selected as a representative pathway for mechanistic validation because it is closely associated with the regulation of inflammatory mediators evaluated in the present study and was linked to several hub genes identified in the pathway–gene network analysis. Notably, this selection does not exclude the potential involvement of other enriched pathways, including MAPK, TNF, IL-17, and Toll-like receptor signaling. The PI3K/Akt pathway plays a central role in inflammatory responses and directly regulates NF-κB activation in response to cytokines such as IL-1β and TNF-α (Ferrara and Mastrocola, 2024). Therefore, inhibiting the PI3K/Akt/NF-κB axis effectively suppresses the expression of inflammatory mediators that drive periodontal tissue destruction. Based on these integrated network pharmacological results, we hypothesized that modulation of the PI3K-Akt-IKK-IκB-NF-κB cascade represents one of the major mechanisms through which PBE exerts therapeutic effects on periodontal inflammation.

To further elucidate the anti-inflammatory and periodontal tissue-protective effects of PBE, we conducted an *in vitro* study using RAW264.7 macrophages and PDLFs. Macrophages play a critical role in pathogen recognition and clearance and in regulating inflammatory amplification and resolution (Wang et al., 2021; Almubarak et al., 2020; Yucel-Lindberg and Bage, 2013). PDLFs, which comprise approximately 50%–60% of periodontal cells, actively participate in inflammatory signaling by releasing prostaglandins, proteolytic enzymes, and bone-modulating factors (Nishikawa et al., 2002; Hormdee et al., 2005). However, under pathological stimuli, sustained macrophage activation and impaired PDLF function cause increased inflammatory cytokine and MMP expression, ECM degradation, and bone resorption, and inhibition of tissue regeneration (Sapna et al., 2014; Luchian et al., 2022; Al-Majid et al., 2018; Jiang et al., 2025). Therefore, modulating the inflammatory response is essential for preventing tissue destruction and promoting periodontal regeneration (Neurath and Kesting, 2024; Mazurek-Mochol et al., 2024; Rai et al., 2010; Rai et al., 2008; Kusano et al., 1998; Song et al., 2019). Our results showed that PBE treatment can suppress NO, PGE2, TNF-α, and IL-1β production in RAW264.7 cells, reducing macrophage-mediated inflammation. IL-1β markedly increased inflammatory cytokine and MMP production in PDLFs, which is consistent with its established role as a key mediator of periodontal tissue destruction (Mazurek-Mochol et al., 2024; Rai et al., 2010). Notably, PBE reduced IL-1β–induced PGE2, IL-6, and IL-1β production and significantly downregulated MMP-3, MMP-8, and MMP-13 expression in a dose-dependent manner. These findings suggest that PBE suppresses inflammatory ECM degradation through downregulation of MMP expression and may contribute to limiting pathological processes associated with periodontal tissue destruction. To validate the mechanism predicted through network pharmacology, we further examined whether PBE modulates PI3K/Akt-mediated NF-κB signaling in PDLFs. Western blotting revealed that PBE significantly attenuated IL-1β–induced phosphorylation of PI3K, Akt, IKK, IκBα, and NF-κB p65. These findings suggest that PBE attenuates inflammatory responses in PDLFs through suppression of PI3K/Akt and NF-κB signaling. The observed reduction in cytokine and MMP expression was accompanied by decreased phosphorylation of PI3K, Akt, IKK, IκBα, and NF-κB p65, supporting the involvement of this signaling axis in the anti-inflammatory effects of PBE. These findings are consistent with the results of previous studies that natural compounds rich in flavonoids and terpenoids effectively inhibit the PI3K/Akt and NF-κB pathways, mitigating inflammation and tissue damage across various inflammatory conditions (Choy et al., 2019; Ge et al., 2022).

Chemical profiling revealed CA, LG, AG, and luteolin 3′-O-glucuronide as major PBE constituents. We next investigated whether these compounds contribute to the biological activities of PBE in periodontitis. Consistent with previous reports, CA alleviated periodontal inflammation in gingival fibroblasts and prevented alveolar bone loss in ligature-induced periodontitis models (Park and Yoon, 2022; Park et al., 2026; Huang et al., 2022; Nishida et al., 2023; Park and Yoon, 2022). *Ixeris dentata* extracts, which are rich in LG, have also demonstrated protective effects in experimental periodontitis models by reducing inflammatory and oxidative stress marker expression and attenuating alveolar bone loss (Lee et al., 2023). The flavonoid glucuronides identified in the present study share a common flavonoid backbone. Flavonoids are promising therapeutic agents for periodontitis due to their anti-inflammatory and tissue-protective effects (Kim et al., 2024; Hashim et al., 2025). Accordingly, we evaluated the anti-inflammatory activities of these major candidate compounds by assessing their ability to suppress the production of inflammatory mediators and cytokines *in vitro*. This approach was essential to verify whether the multi-target interactions predicted using network pharmacology and molecular docking translated into biological efficacy (Nogales et al., 2022; Yuan et al., 2017). Although molecular docking provides valuable insights into potential protein–ligand interactions, docking scores reflect relative binding tendencies rather than absolute binding free energies. Nonetheless, when applied consistently across multiple targets, docking analyses are useful for identifying multi-target interaction profiles, which were further supported by experimental validation in this study (Lee et al., 2020; Pagadala et al., 2017; Meng et al., 2011). Furthermore, docking pose analysis indicated that the major compounds occupied the known catalytic or substrate-recognition regions of iNOS, PTGS2, and MMP3, supporting the functional relevance of the predicted interactions. Although CA exhibited the weakest docking affinity and limited efficacy in cell-based assays, previous studies have reported protective effects in animal models (Park et al., 2026). This discrepancy may be explained by the possibility that natural compounds, including CA, undergo metabolic conversion into bioactive metabolites or interact with multiple biological targets. Furthermore, the physiological environment *in vivo* involves systemic immune regulation, tissue–cell interactions, and pharmacokinetic processes that cannot be fully reproduced in simplified *in vitro* systems (Naveed et al., 2018; Manach et al., 2005).

In contrast, flavonoid glucuronides showed stronger anti-inflammatory activity *in vitro*, indicating a greater capacity to regulate inflammatory signaling. Overall, our findings suggest that flavonoid glucuronides, particularly AG and LG, may represent promising candidate active constituents contributing to the anti-inflammatory effects of PBE. It should be noted that the network pharmacology analysis and compound activity evaluation in this study were limited to four HPLC-identified compounds. Therefore, the findings should be considered hypothesis-generating rather than a comprehensive representation of all bioactive components present in PBE. Since PBE is a complex extract containing numerous additional components, the possibility of contributions from other unidentified constituents cannot be ruled out. To elucidate the precise molecular mechanisms underlying the therapeutic effects of PBE and to identify its principal bioactive constituents, additional *in vivo* studies, bioactivity-guided fractionation, and compound isolation studies will be required.

## Data Availability

The original contributions presented in the study are included in the article/Supplementary Material, further inquiries can be directed to the corresponding author.
