# Supplementary material for: Pimpinella brachycarpa extract attenuates periodontal inflammation by modulating the PI3K/Akt–NF-κB signaling pathway
Source: Front Pharmacol. 2026 Sep 29;17:1902876. doi: 10.3389/fphar.2026.1902876 (PMC13658597; doi:10.3389/fphar.2026.1902876)
Supplement: Supplementary file 1 [file DataSheet1.docx]

**Supplementary Method 1**

For phytochemical analysis, HPLC was performed using a Waters system (Milford, MA, USA) equipped with a quaternary pump, auto-sampler, and photodiode array detector. Separation was achieved on a C18 column (250 × 4.6 mm, 5 μm, Luna, Phenomenex, Torrance, CA) using 0.1% trifluoroacetic (solvent A) and acetonitrile (solvent B) in a gradient elution at a flow rate of 1.0 mL/ min as follows: 0–5 min, 10–10% B; 10–30 min, 15–50% B; 35–40 min, 100-5% B; 40–45 min, 5–5% B. The detection wavelength was 250 nm, the column temperature was maintained at 40 °C, and the injection volume was 10 µL. Chlorogenic acid, luteolin-7-O-glucuronide, apigenin-7-Oglucuronide, and luteolin-3′-O-glucuronide were identified and quantified using reference standards purchased from Sigma-Aldrich (St. Louis, MO, USA). All solvents were purchased from J.T. Baker (Phillipsburg, NJ, USA). PBE was analyzed via HPLC. Through a comparison of the retention time (Rt) and mass spectra with reference standards, we identified three phytochemicals predominantly present in the extract. It was confirmed that the quantity of each compound was 22.7 ± 0.03 mg/g of chlorogenic acid, 7.5 ± 0.08 mg/g of luteolin 7-O-glucuronide, 5.9 ± 0.01 mg/g of apigenin 7-O-glucuronide, and 4.9 ± 0.03 mg/g of luteolin 3′-O-glucuronide.

**Supplementary Method 2**

**LPS-induced periodontitis assays**

Six-week-old male Sprague–Dawley rats were obtained from Koatech (Pyeongtaek, Republic of Korea) and acclimated for 1 week under standard laboratory conditions (temperature 22 ± 2 °C, humidity 55 ± 10%, 12 h light/dark cycle) with ad libitum access to food and water. A total of 30 rats were randomly divided into six experimental groups (n = 5 per group). Periodontitis was induced by local injection of *Porphyromonas gingivalis* LPS (P. gingivalis-LPS, Sigma-Aldrich, USA) into the gingiva surrounding the maxillary first molars. After deep anesthesia with ketamine (75 mg/kg) and xylazine (10 mg/kg) administered intraperitoneally, 1 µL of LPS solution (1 mg/mL in saline) was injected into the lingual gingiva using a Hamilton micro syringe (Hamilton, USA). A second injection of the same volume and concentration was administered 3 days later. In the control group, an equivalent volume of saline was injected instead of LPS. From the day of the first LPS injection, test substances were administered once daily through oral gavage for 14 consecutive days. ISD (100 mg/kg/day, *Insadol®* plus; Dongkook Pharmaceutical Co., Ltd., Seoul, Republic of Korea) was used as a positive control. PBE was administered at doses of 37.5, 75, and 150 mg/kg/day. On day 15, all animals were euthanized, and the maxillae were collected for histological and immunohistochemical analyses.

**Histological analysis and scoring**

Maxillary tissues were fixed in 10% neutral-buffered formalin for 48 h, decalcified in 10% ethylenediaminetetraacetic acid (pH 7.4) for 1 month, embedded in paraffin, and sectioned at 4–5 µm thickness. Paraffin-embedded sections were stained with hematoxylin and eosin (H&E) to evaluate periodontal morphology. Alveolar bone loss was assessed by measuring the vertical distance between the cementoenamel junction (CEJ) and the alveolar bone crest (ABC) at the mesial root of the maxillary first molar using ImageJ software (NIH, USA). Superficial root resorption was defined as scalloped depressions on the root surface and quantified by counting the number of resorption lacunae within a standardized region of interest. The severity of periodontal inflammation was graded on a 0–4 scale according to the histological criteria, considering inflammatory cell infiltration, epithelial integrity, and ABC changes. All histological assessments were performed by two blinded observers, and the mean values were used for statistical analysis.

**Immunohistochemistry**

Immunohistochemical staining for CD45 was performed on paraffin-embedded maxillary sections (4–5 µm) using a primary antibody against CD45 (Abcam, Waltham, MA, USA; 1:200). Immunoreactivity was visualized using a DAB chromogen, and the nuclei were counterstained with hematoxylin. The CD45-positive area was quantified as a percentage of the total region of interest using ImageJ software (NIH, USA).

**Tartrate-resistant acid phosphatase (TRAP) staining**

TRAP staining was performed using a TRACP & ALP Double Stain Kit (Takara, Shiga, Japan) following the manufacturer’s instructions. TRAP-positive multinucleated cells along the alveolar bone surface were identified under a light microscope, and the TRAP-positive area was quantified using ImageJ software (NIH, USA).

**Supplementary Method 3**

**Cell culture**

The RAW264.7 murine macrophage (American Type Culture Collection, Rockville, MD, USA) and human PDLF (CC-7049, Lonza, Tokyo, Japan) were cultured in Dulbecco’s modified Eagle medium containing 10% fetal bovine serum and 1% penicillin/streptomycin (Thermo Fisher Scientific Co. Ltd, Waltham, MA, USA) at 37 ℃ in a humidified atmosphere of 5% CO_2_. The PDLFs were used at passage numbers below 10.

**Cell viability and** **enzyme-linked immunosorbent assay (ELISA)**

RAW264.7 and PDLF cells were seeded at a density of 1 × 10^5^ cells/mL or 1 × 10^4^ cells/mL in a 96-well plate. After 24 h, cells were pretreated with serially diluted PBE (50, 100, or 200 μg/mL), AG, LG, or CA (5~100 μg/mL) for 2 h, and then stimulated with LPS (0.5 µg/mL) or IL-1β (1 ng/mL) for 24 h. Following treatment, culture media were collected for ELISA, and the cell medium was replaced with medium containing 10% cell counting kit-8 and further cultured for 2 h. Cellular activity was measured at 450 nm using a SpectraMax M2 microplate Reader (Molecular Devices, CA, USA). Nitric oxide (NO) levels were determined using the Griess reagent system, and IL-6, IL-1β, TNF-α, PGE2, and MMP-3 were measured using ELISA kits following the manufacturer’s instructions.

**Western blotting**

Proteins were extracted using radioimmunoprecipitation assay buffer and quantified using a Bicinchoninic acid assay kit. Cell lysates containing 15 μg of protein were separated using 10% gels and subsequently transferred onto a polyvinylidene fluoride membrane. The membrane was then blocked using a 5% skim milk solution for 1 h and incubated with primary antibodies overnight at 4 °C. After washing, the membrane was incubated with horseradish peroxidase-labeled secondary antibodies for 1 h at room temperature. Subsequently, they were washed with TBST, and signals were detected using an image analyzer (LAS 4000 mini; GE Healthcare Bio-Sciences, NJ, USA) with enhanced chemiluminescence solution. β-actin served as the internal reference, and relative expression was quantified using the ImageJ software (National Institutes of Health, Bethesda, MD, USA).

**Quantitative reverse transcription polymerase chain reaction**

RNA extraction was performed using the RNeasy Mini Kit (Qiagen, Hilden, Germany) following the manufacturer’s instructions. Reverse transcription of RNA was performed using the iScrip select cDNA Synthesis Kit (Bio-Rad Laboratories, Munich, Germany), and qPCR was conducted using the Power SYBR Green PCR Master Mix (Bio-Rad, Hercules, CA, USA) with CFX Connect Real-Time PCR System (Bio-Rad, Hercules, CA, USA). Relative gene expression levels were represented as ^Δ^Ct = Ct target gene − Ct housekeeping gene (glyceraldehyde-3-phosphate dehydrogenase; GAPDH). Changes in gene expression were calculated using the 2^−ΔΔCT^ method. The sequences of primers constructed were as follows: *GAPDH*: F, 5′- GGATTCCTATGTGGGCGACGA-3′ R, 5′- CGCTCGGTGAGGATCTTCATG-3′; *MMP-3*: F, 5′-CACTCACAGACCTGACTCGGTT-3′ R, 5′-AAGCAGGATCACAGTTGGCTGG-3′; *MMP-8*: F, 5′- GTTCAGCAAGCATTTTCGTT-3′ R, 5′- CACGGAGGACAGGTAGAATG-3′; *MMP-13*: F, 5′- ATGCAGTCTTTCTTCGGCTTAG-3′ R, 5′- ATGCCATCGTGAAGTCTGGT-3′.

**Supplementary Method 4**

**Potential target genes related to chemical structures**

Information regarding four compounds (CA, AG, luteolin 3′-glucuronide, and LG) was retrieved from the PubChem database (<https://pubchem.ncbi.nlm.nih.gov/>; accessed on November 13, 2025).

Target genes related to the compounds were identified using multiple databases including STITCH version 5.0 (<https://stitch-db.org/>; accessed on November 13, 2025), PubChem (<https://pubchem.ncbi.nlm.nih.gov/>; accessed on November 13, 2025), Bioinformatics Analysis Tool for Molecular mechanism of Traditional Chinese Medicine (BATMAN-TCM) 2.0 (<http://bionet.ncpsb.org.cn/batman-tcm/#/home>; accessed on November 13, 2025), SwissTargetPrediction (<https://www.swisstargetprediction.ch/>; accessed on November 17, 2025), TM-MC 2.0 (<https://tm-mc.kr/>; accessed on November 13, 2025), and the SEA Search Server (<https://sea.bkslab.org/>; accessed on November 17, 2025).

Periodontal disease-related genes were collected from the GeneCards database version 5.26 (<https://www.genecards.org/>; updated November 10, 2025; accessed on November 18, 2025). Potential target genes were defined as the overlapping genes between the predicted targets of the four compounds and periodontal disease-related genes. The overlapping target genes were visualized using a Venn diagram generated with Venny 2.1.0 (<https://bioinfogp.cnb.csic.es/tools/venny/>; accessed on November 18, 2025)

**Protein-protein interactions (PPIs)**

The PPI analysis of the potential target genes was performed using interaction data obtained from the STRING database version 12.0 (<https://string-db.org/>; accessed on November 21, 2025). The PPI network was subsequently imported into Cytoscape software version 3.10.3 (<https://cytoscape.org/>; downloaded on May 9, 2025) for network visualization and topological analysis.

**Pathway analysis**

Pathway enrichment analysis was performed using the STRING database version 12.0 (<https://string-db.org/>; accessed on November 21, 2025), the Database for Annotation, Visualization and Integrated Discovery (<https://davidbioinformatics.nih.gov/> updated on September 16 2025; accessed on November 21, 2025), and the Kyoto Encyclopedia of Genes and Genomes (KEGG; <https://www.genome.jp/kegg/>; accessed on November 21, 2025). The enrichment results were visualized using Python (version 3.13)-based scripts as bubble plots, integrating -log10(*p*–value), gene counts, and false discovery rate (FDR) values.

**Molecular docking analysis**

Three-dimensional structures of the target proteins, IL1B, IL6, IL8, iNOS, MMP3, PTGS2, and TNFA, were obtained from the Protein Data Bank (<https://www.rcsb.org/>, accessed on December 12, 2025). Molecular docking simulations were performed using AutoDock Vina (version 1.1.2). The geometric center of each protein was calculated using UCSF Chimera (version 1.19) and used as the center of the initial grid box, allowing unbiased exploration of potential ligand-binding regions. Docking was performed using a fixed grid box size of 26 Å × 26 Å × 26 Å centered on the binding site, and the top 20 poses were ranked based on predicted binding energy values (kcal/mol).
